# Supplementary material for: Child public health interventions for conflict-affected populations: A systematic review
Source: PLOS Glob Public Health. 2025 Dec 30;5(12):e0004880. doi: 10.1371/journal.pgph.0004880 (PMC12753068; doi:10.1371/journal.pgph.0004880)
Supplement: Attachment — Submitted filename: Response to Reviewers.docx [file pgph.0004880.s005.docx]

Response to Reviewers

Editor comments

1. Introduction needs to be strengthened by adding citations on SDGs, UNICEF, UNHCR, Sphere guidelines and other relevant reports
   Thank you, we have added additional text and citations to strengthen the introduction (lines 37-44). We have also updated the statistics (lines 4-5).
2. Key words can be increased and relevant to article
   We have expanded the key words
3. Health needs of this study population- mental and physical issues during humanitarian settings, vulnerabilities due to age such as child abuse, child sexual abuse etc needs elaboration in introduction

Thank you, we have added additional text to provide a brief overview of the risks and needs of children in conflict settings (lines 9-16)

4. Authors also need elaborate on current scenario of humanitarian setting in the world - Gaza etc in introduction
   Thank you for this. We’ve added a paragraph to the introduction to give a global perspective and brief descriptions of the context in Gaza and Sudan (lines 22-36).
5. Please use PRISMA checklist to structure the paper
   We have structured according to the PRISMA checklist.
6. Search words and MESH needs to be mentioned. The mentioned search words do not seem to capture the studies mentioned
   We have added a sentence to direct the reader to the full protocol, which is registered with PROSPERO and included a hyperlink directly to the protocol. (lines 63-64). After receiving this feedback, we also re-ran the searches and found that we retrieved the same titles as when we originally undertook the review.
7. Figure 1 - can be made into multiple tables - physical health needs, mental health needs, social interventions, preventive interventions etc so that the readers can understand clearly (Same classification as in Table 2 can be used)
   Or the studies can be classified into cross sectional, analytical, experimental
   Thank you for this suggestion. We have reorganised Table 1 by study design.
8. A flow chart to depict studies included, excluded, etc will be helpful
   Figure 1 is the flow diagram, presented according to the PRISMA guidelines.
9. Discussion needs to be strengthened from the health systems, applicable for current geopolitical scenario - intersectional coordination and how international global health actors can help in these interventions. Concusions and recommendations, limitations with respect to statistics and robust data analysis tools need to be strong and specific
   Thank you for this feedback. Our aim with this study is to present the current evidence on child public health interventions in conflict settings. The included studies show promise, but there is not enough evidence to support discussion or recommendations on health systems, health system strengthening, or adaptations to humanitarian response in the current geopolitical landscape. For this reason, we have focused the discussion on the available evidence for interventions and made recommendations specific to research and to the need for humanitarian paediatrics expertise.

Reviewer #1: This well-written review explores an important topic and its findings will be of great value to people working in humanitarian paediatrics. The discussion is comprehensive and highlights gaps and areas for action.

Thank you.

A few minor comments:
• The paragraph starting on line 206 includes a description on who is accessing the intervention and when (for study reference 41) and a description of service barriers (for study reference 21) rather than describing the outcomes associated with the interventions. While this is of interest and can help inform service development it is not in keeping with the findings presented elsewhere in the article, which focus on intervention outcomes.

Thank you for highlighting this. These two studies unfortunately did not provide intervention outcomes. We included them in the review because they provided data on social determinants of child and adolescent sexual and reproductive health and patterns of access to care. We have added text to clarify this (line 264).

• The relevance of including ‘inspired by an intervention in Syria where informational leaflets were distributed with bread’ is unclear in the sentence on line 143.
Thank you. We have removed the text.

• Consider adding the study reference to the ‘other telemedicine study’ at the end of the sentence on line 247.
Thank you for flagging this. We have added the study citation (line 291).
